# Associations of Physical Activity and Sedentary Behaviors with Dietary Behaviors among US High School Students

**DOI:** 10.1155/2015/876524

**Published:** 2015-05-25

**Authors:** Richard Lowry, Shannon Michael, Zewditu Demissie, Laura Kann, Deborah A. Galuska

**Affiliations:** ^1^Division of Adolescent and School Health, National Center for HIV/AIDS, Viral Hepatitis, STD, and TB Prevention, Centers for Disease Control and Prevention, Atlanta, GA 30329, USA; ^2^Division of Population Health, National Center for Chronic Disease Prevention and Health Promotion, Centers for Disease Control and Prevention, Atlanta, GA 30341, USA; ^3^Division of Nutrition, Physical Activity, and Obesity, National Center for Chronic Disease Prevention and Health Promotion, Centers for Disease Control and Prevention, Atlanta, GA 30341, USA

## Abstract

*Background*. Physical activity (PA), sedentary behaviors, and dietary behaviors are each associated with overweight and obesity among youth. However, the associations of PA and sedentary behaviors with dietary behaviors are complex and not well understood. *Purpose*. To describe the associations of PA and sedentary behaviors with dietary behaviors among a representative sample of US high school students. *Methods*. We analyzed data from the 2010 National Youth Physical Activity and Nutrition Study (NYPANS). Using logistic regression models which controlled for sex, race/ethnicity, grade, body weight status, and weight management goals, we compared dietary behaviors among students who did and did not meet national recommendations for PA and sedentary behaviors. *Results*. Students who participated in recommended levels of daily PA (DPA) and muscle strengthening PA (MSPA) were more likely than those who did not to eat fruits and vegetables. Students who exceeded recommended limits for television (TV) and computer/video game (C/VG) screen time were less likely than those who did not to consume fruits and vegetables and were more likely to consume fast food and sugar-sweetened beverages. *Conclusions*. Researchers may want to address PA, sedentary behaviors, and dietary behaviors jointly when developing health promotion and obesity prevention programs for youth.

## 1. Introduction

Physical activity (PA), sedentary behaviors, and dietary behaviors are important, yet modifiable, determinants of obesity and other chronic diseases [1–4]. Although participation in PA and sedentary behaviors occur independently [5, 6], research suggests that both types of PA-related behaviors may be associated with dietary behaviors [7, 8]. A recent review of clustering of obesity-related behaviors among children and adolescents found that PA, sedentary behaviors, and dietary behaviors cluster in complex ways that are not well understood [7]. While these three behaviors sometimes cluster in healthy and unhealthy ways, many children and adolescents fall into a mixed category, in which they exhibit both healthy and unhealthy behaviors [5, 7]. A study of clustering of PA, sedentary behaviors, and dietary patterns among European adolescents found that only 18% of adolescents demonstrated consistently healthy and 21% consistently unhealthy behaviors in all three areas [5]. Males were highly represented in the cluster with high levels of moderate-to-vigorous PA (MVPA) and low quality diets, while females were highly represented in the cluster with low levels of MVPA and high quality diets [5]. A review of sedentary behavior and dietary intake among adolescents found that sedentary behavior was associated with a less healthy diet, including higher consumption of fried foods and energy-dense snacks and beverages, and lower consumption of fruits and vegetables; associations with fast food consumption and total caloric, fat, and fiber intake were less consistent [8].

More information on the complex associations of PA and sedentary behaviors with dietary behaviors is needed to inform and better design health promotion and obesity prevention programs for youth. One possible explanation for the inconsistencies in associations of PA and sedentary behavior with food choices is the failure to account for the body weight status and weight management goals of adolescents [7]. Nearly one-third of US high school students are overweight or obese [9]. Approximately 63% of female and 33% of male students report that they are trying to lose weight, while 7% of female and 26% of male students report that they are trying to gain weight [9]. Since PA, sedentary behaviors, and dietary behaviors are each significantly associated with the weight management goals of youth [10, 11], it is possible that weight management goals and perhaps body weight status may confound these associations. In addition, most of the published research connecting sedentary behavior, especially screen time, to unhealthy dietary behaviors has focused on television (TV) viewing time [8]. While the prevalence of US high school students who exceeded the recommended 2 or fewer hours/day of TV viewing time has decreased significantly from 43% in 1999 to 32% in 2013, the prevalence of students who spent more than 2 hours/day playing video or computer games or using a computer for something other than school work has nearly doubled in the past decade, from 22% in 2003 to 41% in 2013 [9]. In view of the recent increase in non-TV screen time, more information is needed on the association between computer/video game (C/VG) use and food choices among youth.

This study extends the current literature by describing the associations of multiple indicators of PA and sedentary behavior with healthy and unhealthy dietary behaviors among a representative sample of US high school students. The strengths of this study include examining these associations while controlling for the body weight status and current weight management goals of students, as well as demographic characteristics. In addition, we tested for interactions to determine whether the associations of PA and sedentary behaviors with dietary behaviors varied by sex, race/ethnicity, grade, body weight status, or weight management goal. Finally, this study included separate measures for TV viewing and C/VG use, which allowed us to examine whether patterns of association with dietary behaviors differed by type of screen time.

## 2. Methods

### 2.1. Study Design

We analyzed data from the National Youth Physical Activity and Nutrition Study (NYPANS), a cross-sectional, school-based study conducted in 2010 by the Centers for Disease Control and Prevention (CDC) to collect information on physical activity and dietary behaviors and the determinants of those behaviors among adolescents. NYPANS used a three-stage cluster-sample design to obtain a nationally representative sample of students in grades 9 through 12 who attend public and private high schools in the 50 states and the District of Columbia. Students completed a self-administered questionnaire in their classrooms during a regular class period in the spring of 2010. The school response rate was 82%, the student response rate was 88%, and the overall response rate was 73%. Usable questionnaires were returned by 11,429 students. A weighting factor was applied to each student record to adjust for nonresponse and oversampling of African American/Black and Hispanic/Latino students. Student participation in the study was anonymous and voluntary, and the parental permission procedures utilized in each sampled school were consistent with local school policies. NYPANS was approved by the study contractor's (ICF Macro) institutional review board.

### 2.2. Measures

Neither PA nor dietary behaviors were normally distributed. Therefore, we analyzed the data in a categorical fashion. Whenever possible, we chose cut-points that were consistent with national health objectives and recommendations.

#### 2.2.1. PA and Sedentary Behaviors

PA and sedentary variables were dichotomized to be consistent with current national health objectives and federal guidelines [1–3]. Daily PA (DPA) was assessed by asking the following: “During the past 7 days, on how many days were you physically active for a total of at least 60 minutes per day? (Add up all the time you spent in any kind of physical activity that increased your heart rate and made you breathe hard some of the time.)” DPA was coded as 7 days versus <7 days. Muscle strengthening PA (MSPA) was assessed by asking the following: “On how many of the past 7 days did you do exercises to strengthen or tone your muscles, such as push-ups, sit-ups, or weight lifting?” MSPA was coded as ≥3 days versus <3 days. TV viewing was assessed by asking the following: “On an average school day, how many hours do you watch TV?” Excessive TV viewing was coded as >2 hours versus ≤2 hours. C/VG use was assessed by asking the following: “On an average school day, how many hours do you play video or computer games or use a computer for something that is not school work? (Include activities such as Nintendo, Game Boy, PlayStation, Xbox, computer games, and the Internet.)” Excessive C/VG use was coded as >2 hours versus ≤2 hours.

#### 2.2.2. Dietary Behaviors

The NYPANS questionnaire included six questions to determine students' fruit and vegetable intake during the 7 days before the survey; students were asked about their consumption of 100% fruit juices, fruit, green salad, carrots, potatoes (not counting French fries, fried potatoes, or potato chips), and other vegetables. Other questionnaire items were used to access intake of sugar-sweetened beverages (SSBs), including intake of regular soda or pop, sports drinks, and energy drinks. The response options for all of these dietary intake questions were as follows: none during the past 7 days; 1 to 3 times during the past 7 days; 4 to 6 times during the past 7 days; 1 time per day; 2 times per day; 3 times per day; and 4 or more times per day (Table 1).

The numbers of times during the past 7 days a student drank 100% fruit juices and ate fruit were summed to determine total fruit intake and then divided by 7 to determine the average daily intake of fruits. The numbers of times a student ate green salad, carrots, potatoes (not counting French fries, fried potatoes, or potato chips), and other vegetables were summed to determine total vegetable intake and then divided by 7 to determine the average daily intake of vegetables. The daily fruit intake variable was dichotomized into ≥2 versus <2 times/day and the daily vegetable intake variable was dichotomized into ≥3 versus <3 times/day to be consistent with the Healthy People 2010 objectives, the national objectives in effect during the study period [12].

The Dietary Guidelines for Americans, 2010, define SSBs as, “Liquids that are sweetened with various forms of sugars that add calories. These beverages include, but are not limited to, soda, fruit ades and fruit drinks, and sports and energy drinks.” Responses to questionnaire items regarding intake of regular soda or pop, sports drinks, energy drinks, and other SSBs were summed to represent total SSB intake and divided by 7 to determine daily SSB intake, and then the variable was dichotomized into ≥3 times/day and <3 times/day. This cut-point was based on a study of Americans aged 2 years and above which found that the estimated 90th percentile of energy intake from SSB on any given day was 450 kcal (equivalent to three 12-oz cans of soda) and was chosen to identify those students at greatest risk from overconsumption of SSB and to match the cut-point used by another published study of SSB intake which uses the NYPANS dataset [13].

In addition to specific dietary intake questions, students were asked about the frequency with which they ate fast food during the past 7 days. When dichotomizing frequency of fast food consumption, the strongest association between the number of fast food meals consumed per week and obesity is when one or more fast food meals are consumed per week [2]. Therefore, we chose to dichotomize this variable into eating fast food ≥1 day/week versus 0 days/week (Table 1).

#### 2.2.3. Demographics, Body Weight Status, and Weight Management Goals (Control Variables)

Demographic variables included sex, race/ethnicity (non-Hispanic white, non-Hispanic black, and Hispanic), and grade (9th, 10th, 11th, and 12th).

Body weight status was based on body mass index (BMI) calculated from measured height and weight. Before measurements were taken, students were asked to remove outer clothing (e.g., coats), purses, shoes, hats, and any removable hair accessories and to remove personal items from their pockets. Based on reference data from growth charts produced by CDC, students with BMI ≥ 95th percentile for sex and age were considered to be obese; students with 85th ≤ BMI < 95th percentile were considered to be overweight; students with 5th ≤ BMI < 85th percentile were considered to be normal weight; and students with BMI < 5th percentile were considered to be underweight.

Weight management goals were assessed by asking the following: “Which of the following are you trying to do about your weight?” Response options were “lose weight,” “gain weight,” “stay the same weight,” and “I am not trying to do anything about my weight.”

### 2.3. Data Analyses

Data were weighted to provide national estimates and analyzed using SUDAAN version 10.0.1 (Research Triangle Institute, Research Triangle Park, NC) to account for the complex sample design. First, we calculated prevalence estimates with 95% confidence intervals (CIs) for body weight status, weight management goals, PA and sedentary behaviors, and dietary behaviors. Bivariate analyses were conducted to identify significant differences in prevalence estimates by sex. Chi-square tests were considered statistically significant if *p* < 0.05. Next, multivariate logistic regression was used to estimate the strength of association (odds ratio, OR) of PA and sedentary behaviors with healthy and unhealthy dietary behaviors. ORs were adjusted for sex, race/ethnicity, grade, body weight status, and weight management goals of students. Since we tested a total of 16 associations, we chose to adjust for multiple comparisons by employing a Bonferroni correction [14]. The correction factor suggested a *p* value of 0.05/16 = 0.003. Therefore, we considered associations of PA and sedentary behaviors with dietary behaviors to be statistically significant if *p* < 0.01. Finally, we tested for interactions (i.e., effect modification) to determine whether the associations of PA and sedentary behaviors with dietary behaviors varied by sex, race/ethnicity, grade, body weight status, or weight management goal. Since we tested a total of 80 possible interactions, we again adjusted for multiple comparisons by employing a Bonferroni correction [14]. The correction factor suggested a *p* value of 0.05/80 = 0.0006. Therefore, we considered interactions by sex, race/ethnicity, grade, body weight status, or weight management goals for any of the 16 associations of PA and sedentary behaviors with dietary behaviors (found in Table 3) to be statistically significant if *p* < 0.001.

## 3. Results

### 3.1. Gender Differences in the Prevalence of Body Weight Status, Weight Management Goals, PA and Sedentary Behaviors, and Dietary Behaviors

Approximately half of students were female (49.4%) and half male (50.6%). Female students were more likely than male students to be obese or underweight, trying to lose weight, and spending time watching TV (Table 2). Male students were more likely than female students to be normal weight, be trying to gain weight, be physically active, use computers/video games excessively, eat fruit and fast food, and drink SSBs (Table 2).

### 3.2. Associations of PA and Sedentary Behaviors with Dietary Behaviors

Participation in DPA and MSPA were significantly and positively associated with consumption of fruits (OR = 2.1 and OR = 1.7, resp.) and vegetables (OR = 2.3 and OR = 1.8, resp.) (Table 3). In addition, DPA was positively associated with consumption of SSB.

Both excessive TV viewing and C/VG use were associated with lower consumption of fruits and vegetables, as well as higher consumption of fast food and SSB (Table 3). Of note, the association of excessive C/VG use with consumption of fast food was not statistically significant when controlling only for demographics (OR = 1.2, *p* = 0.032); however, the association became statistically significant when we included body weight status and weight management goals in the model (OR = 1.3, *p* = 0.005). The statistical significance of the other associations remained unchanged with or without the inclusion of body weight status and weight management goals of students.

### 3.3. Effect Modification of Associations of PA and Sedentary Behaviors with Dietary Behaviors

None of the interactions by sex, race/ethnicity, grade, body weight status, or weight management goals were statistically significant (data not shown). Therefore, the associations of PA and sedentary behaviors with dietary behaviors presented in Table 3 did not vary significantly by sex, race/ethnicity, grade, body weight status, or weight management goals of students.

## 4. Discussion

Participation in PA, sedentary behaviors, and dietary behaviors are known to vary by age, sex, race/ethnicity, and other demographic categories suggesting the need for targeted intervention programs. Our findings suggest that PA-related behaviors and dietary behaviors do not occur in isolation; rather they are strongly associated with each other. The lack of significant statistical interactions in our study further suggests that the associations of PA and sedentary behaviors with healthy and unhealthy dietary behaviors are robust and do not vary by sex, race/ethnicity, grade, body weight status, or weight management goals of students.

In our study, fruit and vegetable consumption was positively associated with DPA and MSPA. These findings are consistent with studies which suggest that more active individuals may be motivated to eat healthier [5, 15]. Other studies, however, suggest that some people may try, consciously or unconsciously, to compensate for unhealthy behaviors in one area of their life by adopting healthy behaviors in another area [5, 16]. This may explain the positive association between DPA and consumption of SSBs found in our study. For example, physically active students may feel that drinking SSBs is not a problem because of the calories they burn during DPA. Also, students with high levels of participation in PA were more likely to consume sports drinks (data not shown).

Sedentary behaviors in the form of TV and C/VG use were consistently associated with lower fruit and vegetable consumption and higher consumption of fast food and SSBs. These findings are consistent with previous studies of TV viewing screen time and dietary intake [8]. Given the recent decrease in TV screen time and increase in C/VG screen time among youth, it is interesting to note that the associations between C/VG use and dietary intake are similar to the associations between TV viewing and dietary intake. Previous studies of advertising and programming content have been confined largely to TV viewing and have documented the presence of frequent advertisements for and references to fast food, SSBs, and other nutrient-poor calorically dense snacks along with a relative lack of advertising for and references to low-calorie, nutrient-dense foods such as fruits and vegetables [17]. In light of the increasing time spent by adolescents using computers online and video games, additional studies are needed to assess the advertising content for healthy and unhealthy foods and beverages found on the Internet and video gaming platforms and the impact that advertising has on dietary behaviors. In addition to the effect of advertising on dietary intake, it may simply be easier to snack on beverage and food items while engaged in sedentary activities than more physically active pursuits. Finally, the fact that controlling for body weight status and weight management goals only changed the statistical significance of one association (i.e., the association between C/VG use and fast food consumption only became statistically significant when body weight status and weight management goals of students were taken into account) suggests that while controlling for these factors may not be crucial when assessing associations of PA and sedentary behavior with dietary behaviors, it may be useful to adjust for these factors when possible. Nearly all of the change from statistical nonsignificance to statistical significance for the association between C/VG use and fast food consumption was the result of adjusting for body weight status, with only a small contribution from adjustment for weight management goals.

This study has several limitations that must be acknowledged. First, these data apply only to youth who attend high school and therefore are not representative of all persons in this age group. The ages of US high school students typically range from 14 to 17 years. Nationwide, in 2009, of persons aged 16-17 years, approximately 4% were not enrolled in a high school program and had not completed high school [18]. Second, these behaviors are self-reported and therefore the extent of underreporting or overreporting could not be determined, although many of the questions analyzed in this study appear on the Youth Risk Behavior Survey (YRBS) questionnaire and psychometric studies have shown that the national YRBS questions demonstrate good test-retest reliability [19]. Thirdly, dietary intake of fruits and vegetables was measured as frequency of consumption without specification of quantity. However, we felt the number of times per day these food items were consumed provided a reasonable indicator of the relative likelihood that students were meeting the Healthy People 2010 national health objectives for consumption of fruits and vegetables. Finally, the data are cross-sectional and therefore causality and directionality of associations cannot be determined.

## 5. Conclusions

PA and sedentary behaviors are each strongly associated with dietary behaviors among US high school students, irrespective of sex, race/ethnicity, grade, body weight category, and weight management goals. It is important that we understand how to address these behaviors simultaneously and in multiple environments to have the best chance of achieving health behavior change in adolescents. A recent review of obesity prevention programs found that programs which combine diet and PA interventions in a school-based setting with home and community components have the most evidence for effectiveness [20].

Schools can be an important venue for health promotion. Creation of a school wellness council or school health team can facilitate the implementation of district wellness policies that promote both healthy eating and PA [21]. A school-wide plan to promote healthy eating and PA might include efforts to create and sustain a healthy school environment by improving physical education, health education, and nutrition programs and encouraging school staff across these areas to work together to coordinate health messages and model healthy behaviors so they are consistent, complimentary, and resource saving.

In addition to school-based programs, efforts to promote healthy eating and PA among youth need to involve the home and community environments. A recent study found that dietary and physical activity behaviors cluster similarly for parents and their children [22]. This suggests that it may be helpful to engage parents so they model and support healthy eating and PA as well as limiting screen time in the home environment. Similarly, the community environment can support advertising and promotional messages that stress the importance of healthy eating and PA, as well as limiting sedentary screen time [23]. Further research is needed to understand how these multiple environments—school, home, and community—can work together and incorporate a variety of approaches to increase healthy eating and physical activity and limit sedentary behaviors.

## Figures and Tables

**Table 1:** Question wording and analytic coding for dietary variables—National Youth Physical Activity and Nutrition Study, 2010.

Dietary behaviors	Questionnaire item(s)	Analytic coding
Fruits	Combination of two items: (1) During the past 7 days, how many times did you eat **fruit**? (Do **not** count fruit juice) (2) During the past 7 days, how many times did you drink **100% fruit juices** such as orange juice, apple juice, or grape juice? (Do **not** count punch, Kool-Aid, sports drinks, or other fruit-flavored drinks)	For combined variable: ≥2 times/day versus <2 times/day (referent)

Vegetables	Combination of four items: (1) During the past 7 days, how many times did you eat green salad? (2) During the past 7 days, how many times did you eat potatoes? (Do not count French fries, fried potatoes, or potato chips) (3) During the past 7 days, how many times did you eat carrots? (4) During the past 7 days, how many times did you eat other vegetables? (Do not count green salad, potatoes, or carrots)	For combined variable: ≥3 times/day versus <3 times/day (referent)

Fast food	During the past 7 days, on how many days did you eat at least one meal or snack from a fast food restaurant such as McDonald's, Taco Bell, or KFC?	≥1 day/week versus 0 days/week (referent)

Sugar-sweetened beverages	Combination of four: (1) During the past 7 days, how many times did you drink a can, bottle, or glass of **soda or pop**, such as Coke, Pepsi, or Sprite? (Do not count diet soda or diet pop) (2) During the past 7 days, how many times did you drink a can, bottle, or glass of a **sports drink** such as Gatorade or PowerAde? (Do** not** count low-calorie sports drink such as Propel or G2) (3) During the past 7 days, how many times did you drink a can, bottle, or glass of an **energy drink** such as Red Bull or Jolt? (Do** not** count diet energy drinks or sports drinks such as Gatorade or PowerAde) (4) During the past 7 days, how many times did you drink a can, bottle, or glass of a **sugar-sweetened beverage **such as lemonade, sweetened tea or coffee drinks, flavored milk, Snapple, or Sunny Delight? (Do** not** count soda or pop, sports drinks, energy drinks, or 100% fruit juice)	For combined variable: ≥3 times/day versus <3 times/day (referent)

**Table 2:** Prevalence (%) of body weight status, weight management goals, physical activity, sedentary behaviors, and dietary behaviors by sex, among US high school students.

Measures	Female		Male		Chi-square test
	%	(95% CI)	%	(95% CI)	(*p* value)
Body weight status^a^					
Obese (BMI ≥ 95th percentile)	20.5	(18.0–23.2)	17.6	(15.9–19.5)	(*p* < 0.05)
Overweight (85th ≤ BMI < 95th percentile)	17.4	(16.0–18.9)	18.2	(16.4–20.2)	
Normal weight (5th ≤ BMI < 85th percentile)	58.9	(56.2–61.6)	62.4	(59.8–64.9)	
Underweight (BMI < 5th percentile)	3.2	(2.4–4.1)	1.8	(1.3–2.5)	
Weight management goals^b^					
Lose weight	60.5	(57.9–63.1)	33.8	(31.6–36.1)	(*p* < 0.001)
Gain weight	5.7	(4.6–7.0)	28.8	(26.7–31.0)	
Stay the same weight	19.0	(17.7–20.4)	19.7	(18.2–21.3)	
Not trying to do anything about weight	14.8	(13.2–16.5)	17.7	(15.9–19.6)	
Physical activity					
Daily physical activity^c^	8.4	(7.3–9.5)	21.7	(19.3–24.4)	(*p* < 0.001)
Muscle strengthening^d^	36.6	(34.1–39.1)	64.4	(60.8–67.9)	(*p* < 0.001)
Sedentary behaviors					
Television viewing^e^	30.2	(26.9–33.8)	26.4	(24.0–29.0)	(*p* < 0.05)
Computer/video game use^f^	19.2	(17.4–21.3)	27.6	(25.4–30.0)	(*p* < 0.001)
Dietary behaviors					
Fruits^g^	39.1	(36.3–42.0)	42.8	(41.2–44.4)	(*p* < 0.05)
Vegetables^h^	18.3	(16.3–20.5)	18.6	(17.3–20.1)	NS
Fast food^i^	72.6	(68.2–76.6)	77.1	(74.3–79.7)	(*p* < 0.01)
Sugar-sweetened beverages^j^	18.1	(15.5–21.1)	26.4	(23.1–30.0)	(*p* < 0.001)

CI = confidence interval. NS = not significant, *p* ≥ 0.05.

BMI = body mass index = weight (kg)/height (m)^2^.

^a^Based on BMI calculated from measured height and weight and using age- and sex-specific percentiles from growth charts developed by Centers for Disease Control and Prevention.

^b^Which of the following are you trying to do about your weight?

^c^Physically active for ≥60 minutes, 7 days/week.

^d^Participated in muscle strengthening exercises on ≥3 days/week.

^e^Watched television for >2 hours on an average school day.

^f^Played video or computer games or used a computer for something other than school work >2 hours on an average school day.

^g^Ate fruits ≥2 times/day.

^h^Ate vegetables ≥3 times/day.

^i^Ate at least one meal or snack from a fast food restaurant ≥1 days/week.

^j^Drank sugar-sweetened beverages ≥3 times/day.

**Table 3:** Prevalence (%) and adjusted odds ratios (ORs) for dietary behaviors by physical activity-related behaviors, among US high school students.

PA-related behavior	Fruits^a^		Vegetables^b^		Fast food^c^		Sugar-sweetened beverages^d^	
	%	OR (95% CI)	%	OR (95% CI)	%	OR (95% CI)	%	OR (95% CI)
Daily physical activity^e^								
Yes	57.8	2.1^‡^ (1.8–2.6)	30.1	2.3^‡^ (1.8–2.8)	73.2	0.9 (0.7–1.0)	29.9	1.4^‡^ (1.2–1.8)
No	38.0	Referent	16.4	Referent	75.1	Referent	20.9	Referent
Muscle strengthening^f^								
Yes	48.0	1.7^‡^ (1.6–1.9)	22.8	1.8^‡^ (1.6–2.1)	74.3	0.9 (0.7–1.1)	23.7	1.0 (0.9–1.2)
No	33.7	Referent	14.1	Referent	75.3	Referent	20.7	Referent
Television viewing^g^								
Yes	36.9	0.7^‡^ (0.6–0.8)	16.4	0.8^†^ (0.6–0.9)	79.8	1.4^†^ (1.1–1.8)	31.0	1.7^‡^ (1.5–2.0)
No	42.5	Referent	19.2	Referent	72.8	Referent	18.7	Referent
Computer/video game use^h^								
Yes	34.1	0.7^‡^ (0.6–0.8)	15.4	0.7^‡^ (0.6–0.9)	78.2	1.3^†^ (1.1–1.6)	29.4	1.6^‡^ (1.3–1.9)
No	43.0	Referent	19.4	Referent	73.8	Referent	20.0	Referent

OR = odds ratio, adjusted for sex, race/ethnicity, grade, body weight status, and current weight management goals.

CI = confidence interval. ^†^ *p* < 0.01.^‡^ *p* < 0.001.

^a^Ate fruits ≥2 times/day.

^b^Ate vegetables ≥3 times/day.

^c^Ate at least one meal or snack from a fast food restaurant ≥1 day/week.

^d^Drank sugar-sweetened beverages ≥3 times/day.

^e^Physically active for ≥60 minutes, 7 days/week.

^f^Participated in muscle strengthening exercises on ≥3 days/week.

^g^Watched television for >2 hours on an average school day.

^h^Played video or computer games or used a computer for something other than school work >2 hours on an average school day.
